# Stapled versus hand-sewn intestinal anastomosis in pediatric patients: a systematic review and meta-analysis

**DOI:** 10.1186/s12887-021-02915-6

**Published:** 2021-10-06

**Authors:** Takayuki Fujii, Aya Tanaka, Hiroto Katami, Ryuichi Shimono

**Affiliations:** Department of Pediatric Surgery, Faculty of Medicine, Kagawa University, 1750-1, Ikenobe, Mikicho, 761-0793 Kitagun, Kagawa Japan

**Keywords:** Functional end-to-end anastomosis, Stapled intestinal anastomosis, Neonates, Infants, Children

## Abstract

**Background:**

The safety and feasibility of stapled intestinal anastomosis have been widely reported in adults. However, the efficacy of stapled anastomosis (SA) in children is unclear. The aim of this study was to perform a systematic review and meta-analysis to evaluate the safety and effectiveness of SA compared with hand-sewn anastomosis (HA) in pediatric patients.

**Methods:**

A systematic literature search was performed using PubMed, the Cochrane Library, and Scopus databases. Studies comparing outcomes of children aged < 7 years and subgroups of children aged < 1 year who underwent SA or HA were included. Primary outcomes were anastomotic leakage and anastomotic stricture. Mean differences (MDs) with 95 % confidence intervals (CIs) were calculated for continuous variables. Odds ratios (ORs) with 95 % CIs were calculated for dichotomous variables. Interstudy heterogeneity was assessed using the chi-squared test and was quantified using the *I*^²^ statistic.

**Results:**

One randomized control trial and five retrospective cohort studies, comprising 633 cases (229 SA cases and 404 HA cases), were included. No significant differences were observed in anastomotic leakage (6.5 % vs. 7.4 %; OR, 0.93; 95 % CI, 0.37–2.34; *p* = 0.88), anastomotic stricture (4.1 % vs. 9.3 %; OR, 0.54; 95 % CI, 0.19–1.51; *p* = 0.24), ileus (7.1 % vs. 9.3 %, OR, 2.35; 95 % CI, 0.15–37.51; *p* = 0.54), anastomosis-related complications (9.5 % vs. 10.9 %, OR, 0.98; 95 % CI, 0.52–1.86; p = 0.96; *I*^2^ = 39 %), and time until full-feeding (MD = -3.57 days; 95 % CI, -11.36 to 4.23; *p* = 0.37) between SA and HA. Operative time was significantly shorter in SA than in HA in children aged < 1 year (MD = -20.36 min; 95 % CI, -26.13 to -14.59).

**Conclusions:**

SA required shorter operative time and was comparable to HA in the overall complication rate. Although the evidence was insufficient, SA could be an option for intestinal anastomosis in children.

## Background

In pediatric surgery, intestinal anastomosis is performed in patients of various ages, conditions, and etiologies such as necrotizing enterocolitis or duodenal atresia (in infants) and Crohn’s disease or malignant lymphoma (in adolescents). A safe and an effective intestinal anastomotic procedure that does not cause complications, including anastomotic leakage, anastomotic stricture, and intestinal obstruction, is required for the rapid recovery of patients.

In adults, stapled anastomosis (SA) is widely used for intestinal anastomosis. A Cochrane review reported that fewer anastomotic leakages are observed with SA than with hand-sewn anastomosis (HA) [1]. This is probably due to less inflammation of the anastomotic site [2], decreased spillage of bowel content during surgery [3], and a uniform method of anastomosis using a stapler [1].

Traditionally, end-to-end HA has been widely used for pediatric patients. Since Powell reported the usefulness of SA in infants younger than 5 months in 1995 [4], reports of SA in pediatric patients have gradually increased [5–12]. These studies reported that SA had the same frequency of complications as HA had [5–11], and SA had a shorter operative time [5, 6, 10, 11], an earlier time until initial feeding [9, 10], and a shorter length of hospital stay than had HA [10]. However, the number of cases in these studies was small, and the effectiveness of SA has not yet been confirmed. Therefore, the aim of this study was to perform a meta-analysis to evaluate the safety and effectiveness of SA compared with HA in pediatric patients.

## Methods

### Protocol registration

This meta-analysis was conducted according to the Preferred Reporting Items for Systematic Reviews and Meta-analyses (PRISMA) guidelines [13]. The protocol was registered in the International Prospective Register of Systematic Reviews (PROSPERO, registration number: CRD42021247302).

### Inclusion and exclusion criteria

Pediatric patients who underwent SA or HA were included. SA was defined as a side-to-side and functional end-to-end anastomosis using any kind of stapling device. HA was defined as an end-to-end anastomosis without using a stapling device. The inclusion criteria were as follows: (1) studies comparing SA and HA, (2) pediatric patients aged < 7 years (to investigate the efficacy and safety of SA in pre-school children), and (3) availability of at least one measurable outcome. Exclusion criteria were: (1) studies in which anastomosis was performed laparoscopically; (2) the article type was an animal study, review, letter, or case report; and (3) duplicate publication or studies that used the same patient group in both studies (in such instances, we chose the most recent study).

### Search strategy

A systematic literature search was performed using PubMed, the Cochrane Library, and Scopus databases for all studies published until May 10, 2021. We placed no limitations on the language of the publication. The following search terms were used: (child* OR pediatric OR paediatric OR infant OR neonate) AND (stapled) AND (anastomo*). The reference lists of the included studies were also reviewed. The search strategy was confirmed by the clinical research expertise of the Clinical Research Support Center at Kagawa University Hospital.

### Data extraction

Data on the characteristics of the study (study design, first author, country and year of publication, sample size, and follow-up period), patients (etiology, age, sex, body weight), anastomosis procedure (the modalities and suturing techniques for anastomosis), and clinical outcomes were extracted. The primary outcomes were anastomotic leakage and anastomotic stricture. The secondary outcomes were operative time, blood loss, postoperative complications (wound infection, abdominal abscess, ileus, and anastomosis related complications requiring reoperation), time until initial feeding, time until full feeding, and length of hospital stay. When there were insufficient data from the published study, we attempted to contact the authors. Data including abstracts or full texts of all potentially relevant studies were independently extracted and evaluated by two independent reviewers (TF and AT).

### Analysis of subgroups

When we obtained at least two studies that sufficiently reported outcomes by subgroups, we conducted a subgroup analysis for patients aged < 1 year.

### Quality assessment

The quality of the included randomized controlled trials (RCTs) was evaluated based on the Cochrane risk-of-bias tool for randomized trials (RoB 2) [14]. The quality of the included non-randomized studies of interventions was evaluated based on the risk of bias in non-randomized studies of interventions (ROBINS-I) tool [15]. Any disagreements were resolved by discussion between the two reviewers or discussion with a third reviewer (RS).

### Statistical analysis

Statistical analysis was performed using Review Manager 5.4 software (Cochrane Collaboration, Oxford, UK). Mean differences (MDs) with 95 % confidence intervals (CIs) were calculated for continuous variables. When the means and standard deviations (SDs) were not available, data for medians and interquartile ranges were extracted and converted to means and SDs using the well-established method reported by Wan et al. [16]. Odds ratios (ORs) with 95 % CIs were calculated for dichotomous variables. The MDs and ORs were considered statistically significant when the *p*-value was < 0.05. Interstudy heterogeneity was assessed using the chi-squared test and was quantified using the *I*^²^ statistic. If the *I*^²^ value was less than 50 %, a fixed-effects model was applied; otherwise, a random-effects model was applied.

## Results

### Study characteristics

**Table 2:** Modalities and suturing techniques for anastomoses

References	Stapled anastomosis	Hand-sewn anastomosis	
	Stapling devices	Suture materials	Suturing techniques
Wrighton [5]	Endo-GIA or GIA linear cutter with 2.5-mm staples (Ethicon Endo-Surgery, Cincinnati, OH, USA)	monofilament or braided, absorbable suture material	-
Kozlov [6]	Endopath ATW-35 Endoscopic Articulating Linear Stapler with 2.5-mm staples (Ethicon Endo-Surgery, Cincinnati, OH, USA)	absorbable suture material	two-layer of continuous
Muncie [7]	30 mm × 2.5 mm endoscopic staplers (Covidien, Mansfield, MA)	5 − 0 silk	single layer of interrupted
Hintz [8]	Proximate, ETS (Ethicon,Somerville, NJ), DST GIA ,and Endo GIA (Covidien New Haven, CT)	monofilament or braided, absorbable suture material (PDS or Vicryl)	-
Amano [9]	Endocutter ETS 35, ETS Flex 45 stapler, and the Echelon Flex Powered ENDOPATH Stapler with 2.5- or 3.5-mm staples (Johnson & Johnson K.K., Tokyo, Japan)	absorbable suture material	-
Mitra [10]	55 mm linear cutting GI stapler	delayed absorbable suture material	single-layer or double-layer

**Fig. 1:**
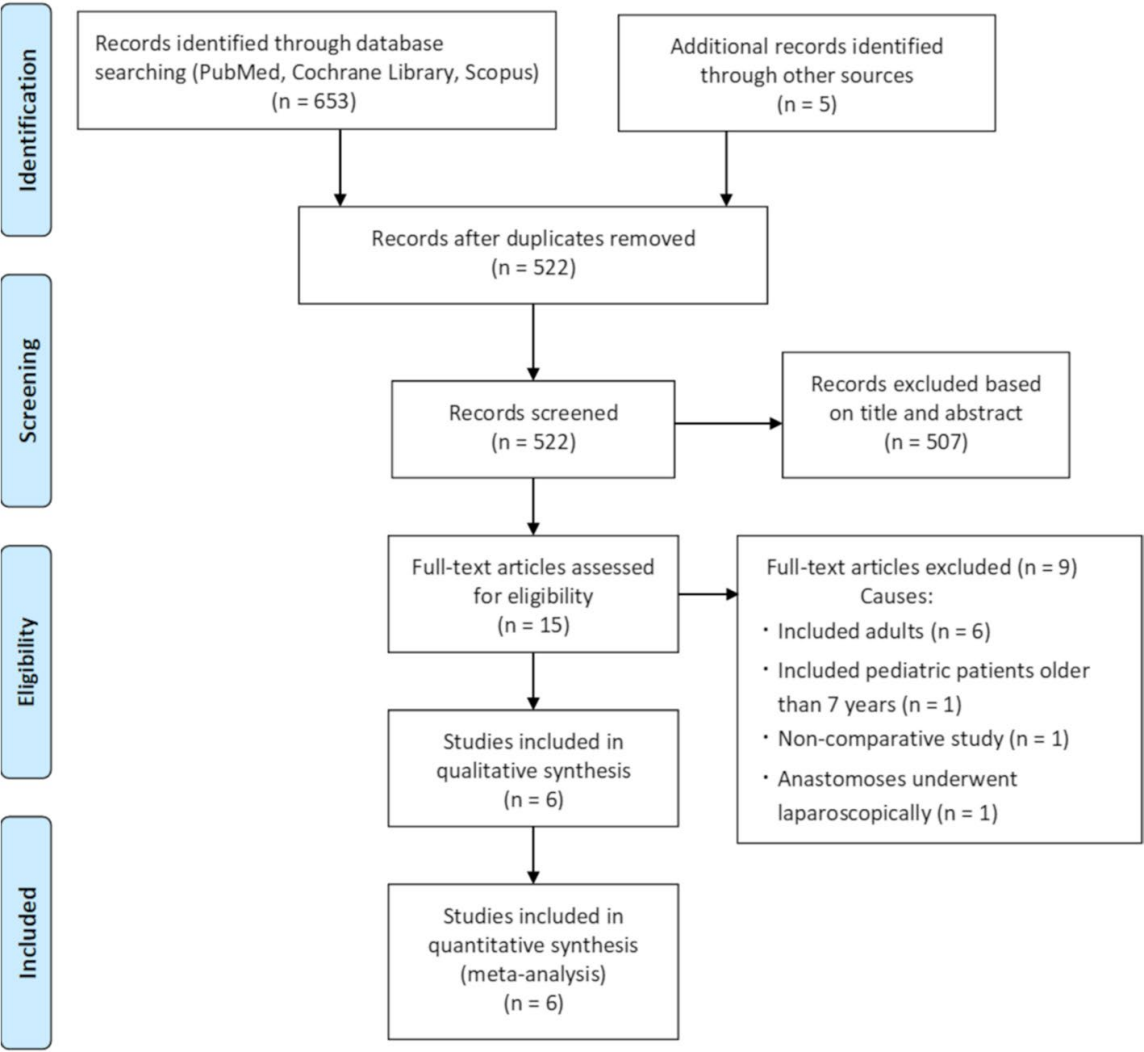
PRISMA flow diagram of this meta-analysis

A total of 522 studies were identified during the initial search (Fig. 1). After title and abstract screening, 15 studies remained. After full-text article screening, nine studies were excluded for the following reasons: included adults (n = 6), included pediatric patients older than 7 years (n = 1), non-comparative study (n = 1), and anastomosis underwent laparoscopically (n = 1). Finally, six studies were included in the meta-analysis [5–10].

Table 1 shows the characteristics of the included studies. The studies included one RCT and five retrospective cohort studies totaling 633 cases: 229 SA cases and 404 HA cases. Except for the RCT study, all cohort studies performed HA when the stapling device was difficult to insert into the intestinal lumen [5–9]. Consequently, patients with HA were significantly younger and had a lighter body weight than those with SA in three studies [5, 7, 9]. Table 2 shows the modalities and suturing techniques used for the anastomoses.

**Table 1:** Characteristics of the included studies

References Author name and year of publication	Country	Study Type	Sample size		Follow-up period	Etiology	Age (days)		Body weight (kg)	
			SA	HA			SA	HA	SA	HA
Wrighton 2008 [5]	USA	R	106	189	up to 9 years	Small intestinal atresia NEC FIP Anorectal malformation with colostomy closure Bowel obstruction Colonic stricture or atresia Bowel perforation Intussusception	105.7	44.5	5.17	3.13
Kozlov 2012 [6]	Russia	R	21	23	averaged 6 months	NEC and FIP Patent omphalomesenteric duct Bowel obstruction Anorectal malformation Gastroschisis Intestinal duplication Hirschsprung disease	23.2	19.7	2.72	2.88
Muncie 2017 [7]	USA	R	38	33	3 months	NEC Small intestinal atresia FIP Gastroschisis Volvulus Bowel ischemia after colostomy Intussusception Perforated small bowel obstruction Anastomotic stricture	≤ 60days (20 %) 61-120days (74 %) > 120days (67 %)	≤ 60days (80 %) 61-120days (26 %) > 120days (33 %)	2.52	2.15
Hintz 2018 [8]	Canada	R	23	67	median of approximately 1 year	Anorectal malformation Intestinal atresia Hirschsprung disease Volvulus Intussusception Meconium ileus Small bowel obstruction Intestinal duplication Gastroschisis Meckel’s diverticulum NEC FIP Dysmotility Familial intrahepatic cholestasis Foreign body ingestion Internal hernia Intestinal stenosis Trauma	10.5 months	8.9 months	8.2	7.95
Amano 2018 [9]	Japan	R	13	64	-	Small intestinal atresia Stoma Meconium peritonitis Volvulus Intestinal duplication Meckel’s diverticulum Intestinal perforation Ileus	2	3	2.8	2.6
Mitra 2020 [10]	India	RCT	28	28	-	Small intestinal atresia Duodenal atresia Intussusception Patent vitellointestinal duct Meckel’s diverticulum Ileostomy closure Total colonic aganglionosis Hirschsprung disease Ileal perforation Gastroschisis Obstructed umbilical hernia	8.89 months	8.39 months	4.11	3.85

*SA* stapled anastomosis, *HA* hand-sewn anastomosis, *R* retrospective cohort study, *RCT* randomized controlled trial, *NEC* necrotizing enterocolitis, *FIP* focal intestinal perforation

### Risk of bias of included studies

**Table 3:** Risk of bias assessment of the included randomized controlled trials (ROB 2)

References	Bias arising from the randomization process	Bias due to deviations from intended interventions	Bias due to Missing outcome data	Bias in measurement of the outcome	Bias in selection of the reported result	Overall Bias
Mitra [10]	Some concerns	Low	Low	Some concerns	Low	Some concerns

Table 3 shows the risk of bias assessment of the included RCTs [10]. There was some concern about bias arising from the randomization process because randomization was performed by allotting patients to each group alternately. In addition, there was some concern about bias arising from the measurement of the outcome because the blinding of outcome assessors was unclear.

**Table 4:** Risk of bias assessment of the included cohort studies (ROBINS-I)

References	Bias due to confounding	Bias in selection of participants into the study	Bias in classification of interventions	Bias due to deviations from intended interventions	Bias due to missing data	Bias in measurement of outcomes	Bias in selection of the reported result	Overall Bias
Wrighton [5]	Serious	Serious	Low	Low	Low	Moderate	Low	Serious
Kozlov [6]	Serious	Moderate	Low	Low	Low	Moderate	Low	Serious
Muncie [7]	Serious	Serious	Low	Low	Low	Moderate	Low	Serious
Hintz [8]	Serious	Moderate	Low	Low	Low	Moderate	Low	Serious
Amano [9]	Serious	Moderate	Low	Low	Low	Moderate	Low	Serious

Table 4 shows the risk of bias assessment in the included cohort studies [5–9]. All studies had a serious risk of bias regarding confounding factors because they included various etiologies, their influence on operative time, and the postoperative outcome cannot be ignored [5–9]. Three studies had a moderate risk of bias in selection of patients because the type of anastomosis performed was determined based on the surgeon’s preference [6, 8, 9]. Another two studies had a serious risk of bias in patient selection [5, 7]. This was because in addition to the decision of anastomosis fashion being determined based upon the surgeon preference, children in the HA group had lower body weight and were younger than those in the SA group, although this was a technical problem [5, 7]. All studies also had a moderate risk of bias in the measurement of outcomes because they were nonblinded studies [5–9].

### Primary outcomes (anastomotic leakage and anastomotic stricture)

**Fig. 2:**
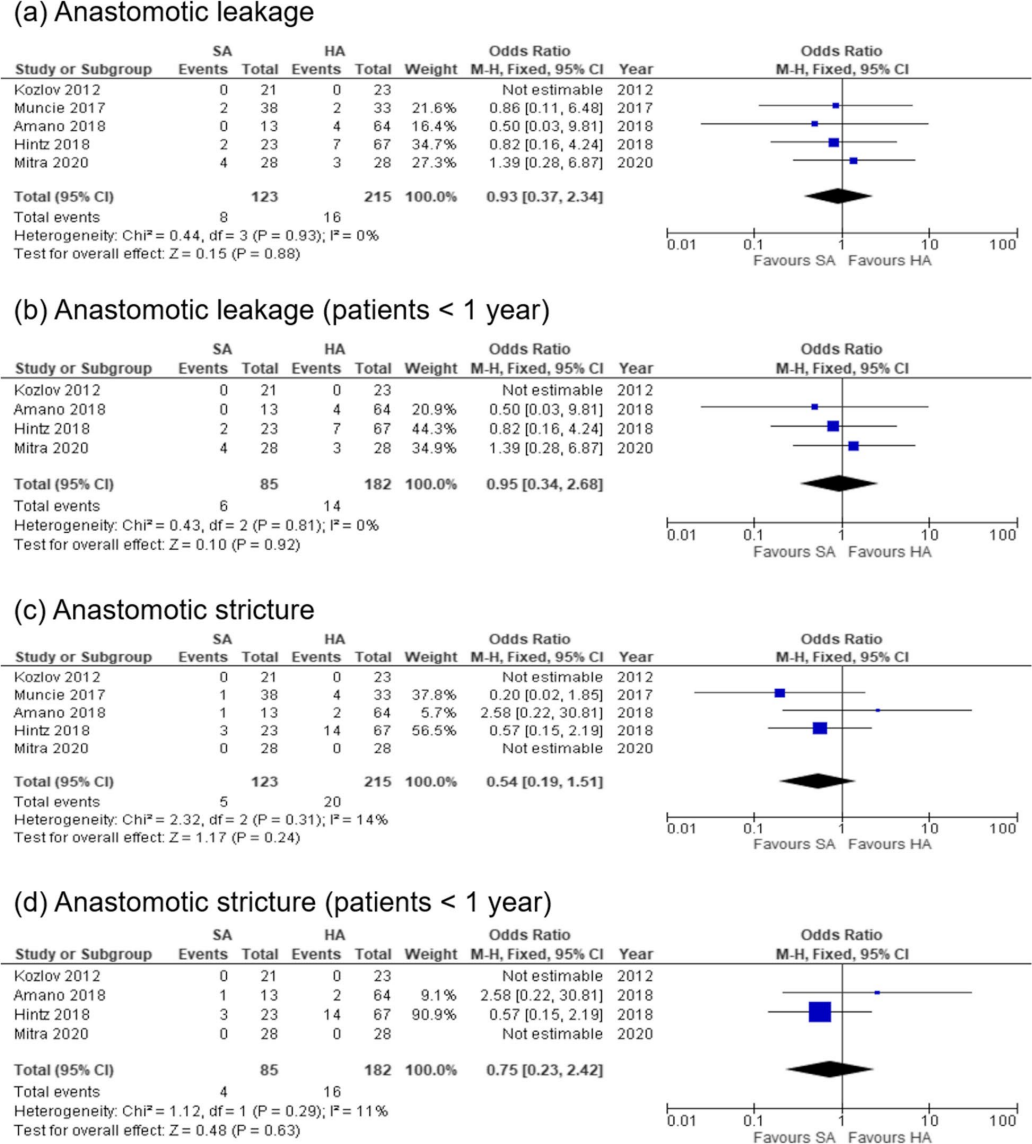
Forest plot displaying the results of primary outcomes

The overall rate of anastomotic leakage was not significantly different between the SA and HA groups (6.5 % vs. 7.4 %, OR, 0.93; 95 % CI, 0.37–2.34; p = 0.88; *I*^2^ = 0 %) (Fig. 2a) [7–10]. In the subgroup analysis of patients aged < 1 year, the overall rate of anastomotic leakage showed no significant difference between SA and HA groups (7.1 % vs. 7.7 %, OR, 0.95; 95 % CI, 0.34–2.68; p = 0.92; *I*^2^ = 0 %) (Fig. 2b) [8–10].

Similarly, the overall rate of anastomotic stricture showed no significant difference between the SA and HA groups (4.1 % vs. 9.3 %, OR, 0.54; 95 % CI, 0.19–1.51; p = 0.24; *I*^2^ = 14 %) (Fig. 2c) [7–9]. In the subgroup analysis of patients aged < 1 year, the overall rate of anastomotic stricture showed no significant difference between the SA and HA groups (4.7 % vs. 8.8 %, OR, 0.75; 95 % CI, 0.23–2.42; p = 0.63; *I*^2^ = 11 %) (Fig. 2d) [8, 9].

### Secondary outcomes

All patients who were assessed for the secondary outcomes were aged < 1 year, with no patients aged ≥ 1 year. Therefore, subgroup analysis was performed for only patients aged < 1 year.

### Ileus

**Fig. 3:**
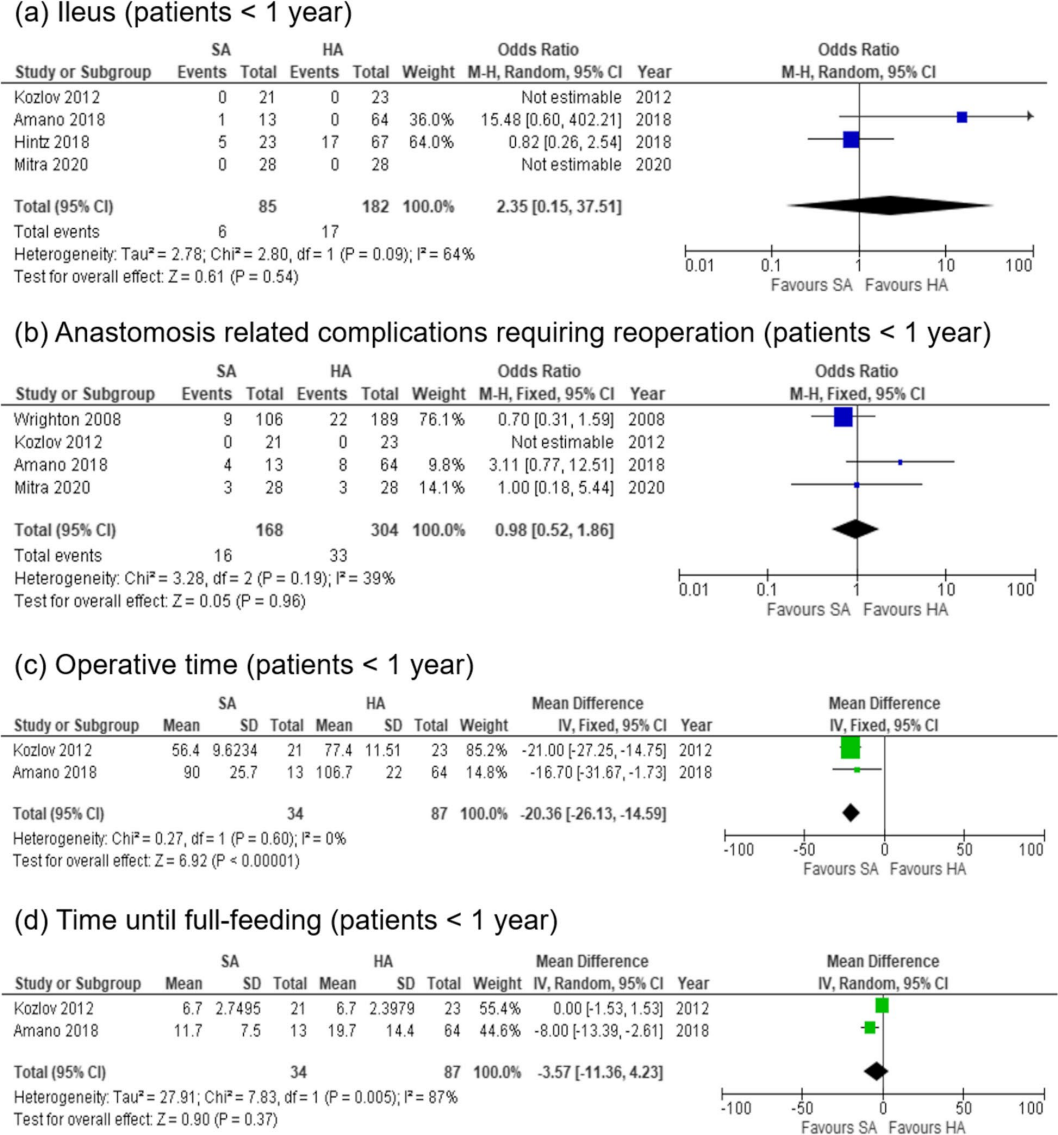
Forest plot displaying the results of secondary outcomes

Because heterogeneity was observed (chi^2^ = 2.8; p = 0.09; *I*^2^ = 64 %), a random-effects model was applied. The overall rate of ileus was not significantly different between the SA and HA groups (7.1 % vs. 9.3 %, OR, 2.35; 95 % CI, 0.15–37.51; p = 0.54) (Fig. 3a) [8, 9].

### Anastomosis related complications requiring reoperation

The overall rate of anastomosis related complications showed no significant difference between the SA and HA groups (9.5 % vs. 10.9 %, OR, 0.98; 95 % CI, 0.52–1.86; p = 0.96; *I*^2^ = 39 %) (Fig. 3b) [5, 9, 10].

### Operative time

Operative time was significantly shorter in the SA group than in the HA group (MD = − -20.36 min; 95 % CI, -26.13 to -14.59; p < 0.001; *I*^*2*^ = 0 %) (Fig. 3c) [6, 9].

### Time until full-feeding

Because heterogeneity was observed (chi^2^ = 7.8; p = 0.005; *I*^2^ = 87 %), a random-effects model was applied. There was no significant difference in the time until full-feeding between the SA and HA groups (MD = -3.57 days; 95 % CI, -11.36 to 4.23; p = 0.37) (Fig. 3d) [6, 9].

**Table 5:** Outcomes of the included studies

References	Operative time (min)		Estimated blood loss (ml)			Wound infection		Abdominal abscess		Time to initial oral feeding (day)		Length of stay (day)	
	SA	HA		SA	HA	SA	HA	SA	HA	SA	HA	SA	HA
Wrighton [5]	102.5	128.2		18.0	18.8	-	-	-	-	-	-	-	-
Kozlov [6]	56.4 (2.1) ^a^	77.4 (2.4) ^a^		-	-	0	0	0	0	-	-	14.1 (1.5) ^a^	13.3 (1.0) ^a^
Muncie [7]	-	-		-	-	-	-	-	-	-	-	-	-
Hintz [8]	127^b^	107^b^	< 10 mL 10–100 mL > 100 mL	34.8 % 52 % 4.3 %	55.2 % 31.3 % 1.5 %	-	-	0	0	7^b^	3^b^	11^b^	7^b^
Amano [9]	85 ^c^ (77–108)	109 ^c^ (91–120)		13 ^c^ (8–24)	10 ^c^ (5–25)	0	0	0	0	4 ^c^ (3–7)	7 ^c^ (5–10)	-	-
Mitra [10]	23.9	33.1		22.7	27.8	2	5	-	-	5.4	7	6.7	8.7

*SA* stapled anastomosis, *HA* hand-sewn anastomosis, ^a^ mean (standard error), ^b^ median, ^c^ median (interquartile range)

The review studies were not enough to evaluate the data on blood loss, wound infection, abdominal abscess, time until initial feeding, and length of hospital stay. The data for each study are listed in Table 5.

## Discussion

The results of this meta-analysis showed that SA was comparable to HA in the overall rate of anastomotic leakage, anastomotic stricture, ileus, and anastomosis related complications in children aged < 1 year. Moreover, the operative time was significantly shorter in the SA group than in the HA group in this population.

The safety and efficacy of SA in adults has been widely reported [1, 17]. A Cochrane review including seven RCTs with 1125 patients reported that SA had a significantly lower incidence of anastomotic leakage than had HA [1]. In addition, a recent network meta-analysis including 11 trials with 1113 patients reported that SA showed a higher probability of superiority to HA in reducing the incidence of overall postoperative complications, clinical recurrences, and reoperation [17]. Therefore, the authors advocated that SA would probably be the optimal anastomosis for Crohn’s disease.

There are few reports about SA in children, which may be due to concerns about the difficulty and safety of inserting the device into the narrow intestinal lumen of young children. However, with the development of minimally invasive surgeries and the compactness of stapling devices, SA can be safely performed, even in newborns and infants. In 1995, Powell reported the usefulness of SA in a case series of seven neonates or young infants with a mean age of 72 days and a mean weight of 3.7 kg [4]. Subsequently, a retrospective cohort study of 44 children with a mean age of 23 days and a mean weight of 2.7 kg in the SA group reported that there were no intra- and postoperative complications compared to those with HA [6]. A recent RCT study of 56 children with a mean age of 8.9 months and a mean weight of 4.1 kg in the SA group reported that there was no difference in the number of complications between the SA and HA groups. Patients with SA had less blood loss and shorter length of hospital stay than that had those with HA [10]. In addition, a multivariate analysis revealed that both SA procedure and body weight were not independent predictors of anastomotic complications [8]. In this study, there were no significant differences in the number of anastomotic leakages, anastomotic strictures, ileus, and anastomosis-related complications requiring surgery in children aged < 1 year. Although there is a limit on the size of the intestinal lumen into which the stapling device can be safely inserted, SA could be acceptable even in small children. Recently, the feasibility and safety of a 5-mm stapler in small children have been reported [18, 19]. Although the 5-mm stapler was not used in this study, such miniature devices may offer an alternative to HA in the smaller intestinal lumen.

Many studies on SA have reported that the operative time was shorter in SA than in HA [5, 6, 9, 10]. Similarly, in this study, operative time was significantly shorter in the SA group than in the HA group in children aged < 1 year. Shorter operative times may reduce the strain on the patient and intestinal tissue and accelerate the recovery of intestinal peristalsis. In fact, in the studies of Mitra et al. and Amano et al., SA had a shorter operative time and lesser time until initial feeding than HA [9, 10].

There were two studies which reported specific complications associated with SA. Jackson et al. reported that children aged 5-and 7 years had intestinal volvulus and bacterial overgrowth due to huge dilatation of the anastomotic site after SA at 2 months and 3 years of age, respectively [20]. Amano et al. reported two cases of complications [9]. One patient was a 5-year-old child who had small intestinal volvulus, with two adhesive bands and anastomotic dilatation after SA at 1 day of age. The other case was an infant who had midgut malrotation with volvulus and anastomotic dilatation 4 months after SA for repair of intestinal atresia. Although the authors believe that the volvulus was mainly caused by malrotation and adhesive bands, it might be triggered by dilatation of the anastomotic site [9]. It should be noted that such intestinal obstruction may occur infrequently.

This study had several limitations that could affect generalization. First, the number of studies identified was limited, which may have resulted in insufficient experience to detect outcomes. Second, the available evidence had a risk of bias because the RCTs had some concerns, and all other cohort studies had a serious risk of bias. In particular, because all studies included various etiologies, their influence on operative time and postoperative outcome cannot be ignored. In addition, all cohort studies had a moderate or serious risk of bias in selection patients because the decision of anastomosis fashion was determined based on the surgeon’s preference or a technical problem. Consequently, the HA groups tended to be smaller and younger than the SA group, which would affect the outcomes. Third, many of included studies had short or unclear follow-up periods. As a result, relatively delayed complications specific to SA, such as bleeding at the staple line [7] or intestinal obstruction, as stated above, may have been overlooked [9, 20]. Despite these limitations, this study was the first meta-analysis to investigate the efficacy of SA in pediatric patients and showed that SA could be comparable to HA. To strengthen our understanding and resolve the problems we faced, a prospective well-designed RCT with a larger number of subjects should be performed.

## Conclusions

In this study, SA had the advantage of a shorter operative time than had HA in children aged < 1 year. SA was comparable to HA in the overall rate of anastomotic leakage, anastomotic stricture, ileus, and anastomosis-related complications. Although the evidence was insufficient, SA could be an option for intestinal anastomosis in a variety of etiologies when inserting the stapling device into the intestinal lumen, even in neonates or infants.

## Data Availability

All data analysed during this study are included in this published article.

## Abbreviations

SA: Stapled anastomosis
HA: Hand-sewn anastomosis
RCT: Randomized controlled trial
MD: Mean difference
CI: Confidence interval
SD: Standard deviation
OR: Odds ratio
